# Developmental regulation of ecdysone receptor (*EcR*) and EcR-controlled gene expression during pharate-adult development of honeybees (*Apis mellifera*)

**DOI:** 10.3389/fgene.2014.00445

**Published:** 2014-12-22

**Authors:** Tathyana R. P. Mello, Aline C. Aleixo, Daniel G. Pinheiro, Francis M. F. Nunes, Márcia M. G. Bitondi, Klaus Hartfelder, Angel R. Barchuk, Zilá L. P. Simões

**Affiliations:** ^1^Departamento de Genética, Faculdade de Medicina de Ribeirão Preto, Universidade de São PauloSão Paulo, Brazil; ^2^Faculdade de Ciências Agrárias e Veterinárias, Universidade Estadual PaulistaSão Paulo, Brazil; ^3^Departamento de Genética e Evolução, Centro de Ciências Biológicas e da Saúde, Universidade Federal de São CarlosSão Carlos, Brazil; ^4^Departamento de Biologia, Faculdade de Filosofia, Ciências e Letras de Ribeirão Preto, Universidade de São PauloSão Paulo, Brazil; ^5^Departamento de Biologia Celular, Molecular e de Bioagentes Patogênicos, Faculdade de Medicina de Ribeirão Preto, Universidade de São PauloSão Paulo, Brazil; ^6^Laboratório de Biologia Animal Integrativa, Departamento de Biologia Celular, Tecidual e do Desenvolvimento, Instituto de Ciências Biomédicas, Universidade Federal de AlfenasAlfenas, Brazil

**Keywords:** honey bee, adult development, 20E, ecdysteroid, juvenile hormone, JH, RNAi, miRNA

## Abstract

Major developmental transitions in multicellular organisms are driven by steroid hormones. In insects, these, together with juvenile hormone (JH), control development, metamorphosis, reproduction and aging, and are also suggested to play an important role in caste differentiation of social insects. Here, we aimed to determine how *EcR* transcription and ecdysteroid titers are related during honeybee postembryonic development and what may actually be the role of EcR in caste development of this social insect. In addition, we expected that knocking-down *EcR* gene expression would give us information on the participation of the respective protein in regulating downstream targets of EcR. We found that in *Apis mellifera* females, EcR-A is the predominantly expressed variant in postembryonic development, while EcR-B transcript levels are higher in embryos, indicating an early developmental switch in EcR function. During larval and pupal stages, EcR-B expression levels are very low, while EcR-A transcripts are more variable and abundant in workers compared to queens. Strikingly, these transcript levels are opposite to the ecdysteroid titer profile. 20-hydroxyecdysone (20E) application experiments revealed that low 20E levels induce *EcR* expression during development, whereas high ecdysteroid titers seem to be repressive. By means of RNAi-mediated knockdown (KD) of both EcR transcript variants we detected the differential expression of 234 poly-A^+^ transcripts encoding genes such as CYPs, MRJPs and certain hormone response genes (*Kr-h1* and *ftz-f1*). EcR-KD also promoted the differential expression of 70 miRNAs, including highly conserved ones (e.g., miR-133 and miR-375), as well honeybee-specific ones (e.g., miR-3745 and miR-3761). Our results put in evidence a broad spectrum of EcR-controlled gene expression during postembryonic development of honeybees, revealing new facets of EcR biology in this social insect.

## Introduction

Most multicellular organisms go through developmental transitions that enable them to cope with environmental changes and/or broaden their niche possibilities. Such transitions are generally timed and synchronized by morphogenetic hormones in a broad range of species, including insects, amphibians, metamorphic fish, tunicates, echinoderms, and plants. In insects, developmental transitions, such as larval and metamorphic molts, are driven by steroid hormones (ecdysteroids) acting in conjunction with juvenile hormone (JH). These hormones also control reproduction and aging (Flatt et al., 2005; Gáliková et al., 2011), and, in social insects, play important roles in caste polyphenism (Hartfelder and Emlen, 2012).

The steroid hormone ecdysone is produced by the prothoracic glands. After secretion, it is transported via the hemolymph to its target organs. Due to its lipophilic nature it passes directly into the cytoplasm of target and/or modification center cells (Iga and Kataoka, 2012; Ono, 2014), where it can be modified to 20-hydroxyecdysone (20E) by a 20-monooxygenase encoded by the *shade* gene, a member of the cytochrome P450 family (CYP314a1) known as Halloween (Petryk et al., 2003). The mode of action of JH, which is a sesquiterpenoid morphogenetic molecule, has only recently become clear (for review see Bellés and Santos, 2014), both in terms of its receptor and downstream cascade, as well as its molecular interaction with ecdysteroids. Produced by the *corpora allata* in the retrocerebral complex, JH relies on binding proteins for its transport in the hemolymph to target cells. There, it first binds to its intracellular receptor, the Methoprene-tolerant (Met) protein, which then forms a complex with Taiman (Charles et al., 2011). This dimeric hormone-receptor complex then regulates the expression of target genes.

Knowledge on the mechanism of action of insect ecdysteroids initiated with the early work of Clever and Carlson (for a historical review see Bellés and Santos, 2014), which eventually resulted in the so-called Ashburner model (Ashburner et al., 1974), which proposed a general model for the action of ecdysone, based on its participation in the regulation of gene expression (puffing) in the polytenic salivary gland chromosomes during *Drosophila melanogaster* molting and metamorphosis. Briefly, the model states that ecdysone associates with an intracellular receptor protein to activate early genes encoding transcription factors, which then activate late genes and, on the other, inhibit the transcription of previously activated early genes. The receptor protein and certain other members of this cascade belong to a large family of proteins, the nuclear hormone receptors (NR, see Fahrbach et al., 2012). NR proteins are generally comprised of four independent but functionally interacting domains. A/B is a highly variable domain that may contain a motif (AF-1) driving ligand-independent transcription. The second, the C domain, is a DNA-binding domain (DBD), the most conserved region of NRs. The D or hinge domain provides a link between DBD and the next domain, LBD, a multifunctional domain that mediates ligand binding, dimerization, and interaction with heat shock proteins, nuclear localization, and transactivation functions. Functional NRs form homodimers and/or heterodimers that recognize specific DNA sequences. In the absence of a ligand molecule they act as repressors maintaining target genes inhibited by co-repressor complexes. In the presence of hormone they are activators of target genes by recruiting co-activator proteins and displacing co-repressors (Hill et al., 2013; Yamanaka et al., 2013; Evans and Mangelsdorf, 2014).

The functional ecdysone receptor is a heterodimeric NR formed by the *Ecdysone Receptor* (*EcR*) and the *ultraspiracle* (*usp*) gene products (for a comprehensive review, see Hill et al., 2013). USP is an ortholog of the vertebrate retinoid-X receptor (RXR) (Yao et al., 1992) and is most commonly considered a kind of orphan NR. Though its ligand is not known, its participation as a mediator of JH action has been postulated, probably through a direct binding of JH (Barchuk et al., 2004). Furthermore, the EcR/USP complex can also bind to the *let-7-C* gene after a 20E pulse has triggered the larval-to-pupal metamorphic molt, thus inducing the transcription of a cluster of three microRNAs (miR-100, let-7 and miR-125). These then post-transcriptionally regulate the expression of genes involved in neuromuscular morphogenesis, leading to adult body characteristics (Chawla and Sokol, 2012; see also Rubio and Bellés, 2013). The EcR protein can also *per se* regulate the expression of target genes (Davis and Li, 2013), thus adding extra levels of complexity to the mechanisms and gene regulatory networks involving hormone/transcription factor activities.

In insects showing caste polyphenism, there is evidence that ecdysteroids are important players in caste differentiation, not only during post embryonic, but possibly even during embryonic development (Schwander et al., 2008). The role of ecdysteroids in caste development and regulation of adult reproduction is currently best understood in bees, especially so in the bumblebee *Bombus terrestris* (Geva et al., 2005) and in the honeybee *Apis mellifera* (Hartfelder and Engels, 1998), where they participate in the regulation of the differential morphogenesis programs by interacting with JH and possibly other mediating environmental modulators.

Receptor proteins mediating ecdysteroids action in social insects have been studied mainly in the honeybee (The Honey Bee Genome Sequencing Consortium, 2006; Velarde et al., 2006), where USP and EcR cDNAs have been cloned (Barchuk et al., 2004; Takeuchi et al., 2007), and the expression profiles of the respective genes were determined in several organs, tissues, and conditions (Barchuk et al., 2004, 2008; Takeuchi et al., 2007; Velarde et al., 2009). However, and despite all these works, several responses to differential hormone signaling in honeybee caste development are still poorly understood (Barchuk et al., 2007). For instance, ecdysteroid titers in developing females are higher in queens during the second half of the last larval instar (Rachinsky et al., 1990) and differ in their profiles during pupal and pharate-adult development of queens and workers (Pinto et al., 2002). These hormone titer differences are associated with the differential development of specific structures (e.g., brain and ovary, Barchuk et al., 2007) and also the onset of vitellogenin synthesis (Barchuk et al., 2002), but this is essentially correlative information lacking functional support. Herein we aimed at determining the extent to which *EcR* transcription follows ecdysteroids titers during honeybee postembryonic development and can actually mediate the action of molecular determinants of caste development in honeybees. Moreover, we expected that knocking-down *EcR* gene expression during pharate-adult development would bring to light new downstream targets of EcR.

## Materials and methods

### Bees

Embryos and the successive developmental phases in the larval and pupal stages, as well as newly-emerged adults were obtained from *A. mellifera* colonies (Africanized hybrids) maintained at the Experimental Apiary of the University of São Paulo at Ribeirão Preto, Brazil. The developmental phases of workers and queens (Table 1) were identified according to Rembold (1987) and Michelette and Soares (1993). Immediately after sampling, the bees were immersed in TRIzol reagent (Life Technologies) and frozen at −80°C until RNA extraction.

**Table 1:** **Developmental stages (embryonic, larval, pupal, adult) of *A. mellifera* considered in this work**.

**Abbreviation**	**Developmental stage**
E	Embryo
L1	First instar larva
L2	Second instar larva
L3	Third instar larva
L4	Fourth instar larva
L5F1	Fifth instar larva, feeding phase 1
L5F2	Fifth instar larva, feeding phase 2
L5F3	Fifth instar larva, feeding phase 3
L5S1	Fifth instar larva, cocoon-spinning phase 1
L5S2	Fifth instar larva, cocoon-spinning phase 2
L5S3	Fifth instar larva, cocoon-spinning phase 3
PP1	Fifth instar larva, prepupa 1
PP2	Fifth instar larva, prepupa 2
PP3	Fifth instar larva, prepupa 3
Pw	White-eyed pupa, unpigmented cuticle
Pp	Pink-eyed/pharate-adult transition, unpigmented cuticle
Pdp	Dark pink-eyed pharate-adult, unpigmented cuticle
Pb	Brown-eyed pharate-adult, unpigmented cuticle
Pbl	Brown-eyed pharate-adult, light pigmented cuticle
Pbm	Brown-eyed pharate-adult, intermediary pigmented cuticle
Pbd	Brown-eyed pharate-adult, dark pigmented cuticle
NE	Newly emerged adult

### Northern blot analysis

Approximately 15 μg of total RNA extracted from queens and workers at the PP1 and Pb developmental stages were subjected to electrophoresis in a denaturing 1.5% agarose/formaldehyde gel, and the RNA was then transferred to a PVF (Polyvinylidene Fluoride, GE) membrane using a VacuGene XL Vacuum Blotting system (GE Healthcare). An EcR cDNA fragment of 160 bp encoding the 3′ part of the DNA-binding domain was used for probe synthesis by means of the Random Primers DNA Labeling System (Life Technologies) and Redivue ^32^P-nucleotides (Amersham). After 3 h of hybridization at 42°C, the membranes were washed during 20 min with 0.1 × SSC solution containing 0.1% SDS and then exposed to a Super Sensitive ST film and the bands revealed with Cyclone™ Storage Phosphor System (PerkinElmer).

### Hormone treatments

For the analysis of the *EcR* expression response to artificially augmented levels of hormones, workers at the brown-eyed pupal phase (Pb) were removed from the brood frames and maintained in an incubator at 34°C and 80% relative humidity. For the ecdysone response, three groups of 3–7 workers were injected with 5 μg of 20-hydroxyecdysone (20E; Sigma) dissolved in 2 μL Ringer saline containing 12.5% ethanol. For the JH response, a similar number of Pb-phase workers received a topical application of 10 μg JH-III (Fluka) dissolved in 2 μL acetone. Controls received 2 μL of the respective solvents. The amounts of applied hormone were based on previous experiments in which we had examined their effects on inducing gene expression during pupal stage (Barchuk et al., 2002, 2004). RNA was isolated from fat bodies after 1, 12, and 24 h (independent experiments). Fat bodies were obtained via a longitudinal incision in isolated abdomens, which were then kept under gentle agitation in Petri dishes containing 0.9% NaCl. The resultant suspension of dispersed fat body cells was centrifuged during 1 min at 2500× g and the pellet was transferred into TRIzol reagent and frozen at −80°C until RNA extraction. We used fat bodies because this allowed us to specifically assay this metabolically important organ, especially with regard to vitellogenin (*vg*) gene expression in honeybees.

### RNA extraction, reverse transcription and quantitative PCR assays

Total RNA was isolated using TRIzol (Life Technologies), following the manufacturer's protocol, and purified by column purification (RNeasy Mini Kit, QIAGEN), as described previously (Barchuk et al., 2004, 2007). For the quantification of mRNA levels (except those validating the RNA-Seq data), first strand cDNA was synthesized by reverse transcription from 2 μg of RNA with SuperScript II Reverse Transcriptase (Life Technologies) and an oligo(dT)_12–18_ primer (Life Technologies). For the validation of the RNA-Seq libraries, cDNA was synthesized using NCode™ miRNA First-Strand cDNA Synthesis and qRT-PCR (Invitrogen) kits and their instructions, adding a DNase (Promega) treatment step.

Comparative analyses of transcript levels were performed by Real Time quantitative PCR (qPCR) using a 7500 Real-Time PCR System (Applied Biosystems) or a StepOne Plus system (Applied Biosystems). Amplifications were carried out in 20 μL reaction mixtures, each containing 10 μL of SYBR® Green Master Mix 2× (Applied Biosystems), 0.8 μL of a 10 mM stock solution of each of the gene-specific forward and reverse primers (Table S1), and 1 μL of first-strand cDNA diluted 1:4 (or 1:10, for cDNA samples used to validate RNA-Seq data) in ultrapure water. The sequences of forward primers were identical to the mature miRNA sequences available at miRBase, but replacing U by T, while the reverse Universal qPCR primer is supplied by NCode kit. Reaction conditions were 50°C for 2 min, 95°C for 10 min, followed by 40 cycles of 95°C for 15 s and 60°C for 1 min (or 33 s for miRNA amplification). Three biological replicates were run in three technical replicates each. *Actin related protein* 1 (*Arp1*, GenBank accession number NM_001185145.1), *rpl32* (accession number NM_001011587.1), or a *U5 snRNA* gene were used as reference genes (for confirmation, cDNAs for all three reference genes were partially sub-cloned and sequenced in our laboratory). Relative quantities of transcripts were calculated using the comparative Ct method (Applied Biosystems, User bulletin#2). Statistical analyses were carried out with Statistica version 7.0 (http://statistica.software.informer.com/).

### Assessing gene transcription patterns associated to EcR function during honeybee development using RNAi

#### dsRNA synthesis and treatment

We employed a general protocol for dsRNA synthesis and injection in honeybees (Pbl phase) (Amdam et al., 2003). For *EcR* dsRNA synthesis, a 391 bp clone of *EcR* cDNA was amplified to serve as template, this comprising a fragment shared by the two transcript variants (A and B). The primers with the respective recognition site for T7 RNA polymerase (underlined) were: *EcR*-forward 5′-TAATACGACTCACTATAGGGCGAGAATGGCGAGGAAGTACGAC and *EcR*-reverse 5′-TAATACGACTCACTATAGGGCGATTCTTGAACTTGAGGCTGAAG. A green fluorescent protein (*GFP*) gene clone was used as template to synthesize the respective dsRNA used as a non-target control (*GFP*-forward 5′-TAATACGACTCACTATAGGGCGAAGTGGAGAGGGTGAAGGTGA-3′ and *GFP*-reverse 5′-TAATACGACTCACTATAGGGCGAGGTAAAAGGACAGGGCCATC-3′; see Nunes et al., 2013a). The amplification products were visualized and retrieved after agarose gel electrophoresis and purified using QIAquick™ (QIAGEN) columns. *In vitro* transcription reactions were performed by using the RiboMax™ T7 system (Promega) and the obtained dsRNA was isolated using TRIzol LS reagent (Invitrogen), subjected to a denaturation step at 98°C for 5 min, followed by 30 min at room temperature, and diluted with nuclease free water to a final concentration of 2.5 μg/μL. The dsRNA quality was assessed by agarose gel electrophoresis.

Pbl-phase workers (*n* = 30 for each experimental group) received an intra-abdominal injection of 2 μL of *EcR* dsRNA solution (2.5 μg/μL). Controls of the same developmental phase received the same volume of *GFP* dsRNA solution. dsRNA-injected bees were kept in an incubator at 34°C and 80% relative humidity until adult eclosion (~2 days), when they were transferred to TRIzol reagent (Invitrogen) and frozen at −80°C until RNA extraction. Total RNA extraction and first strand cDNA synthesis were carried out as described above. *EcR* knockdown efficiencies were assessed by RT-qPCR using variant-specific primers (EcRA-F, EcRB-F, and EcRA/B-R; see Table S1). Bees not used for gene expression analysis were used for evaluation of the adult phenotype.

#### Analysis of gene expression patterns by RNA-Seq

RNA pools of equal concentration from each group of EcR- and GFP-dsRNA treated bees were used for RNA-sequencing. Libraries were prepared using the TruSeq RNA™ Sample Preparation kit (Illumina) for poly-A^+^ RNA, and the TruSeq™ Small RNA Sample preparation kit (Illumina) for small RNAs (shorter than 200 nt). These libraries were shipped to the University of North Carolina (Chapel Hill, USA) facility where they were sequenced on an Illumina platform (Genome Analyzer II, Life Sciences).

RNA-Seq reads for the poly-A^+^ RNA library were first submitted to adapter clipping using Scythe (Buffalo, 2011) (v.0.981—default parameters) for the 3′-end adapter and CutAdapt (Martin, 2011) (v.1.1—minimum overlap of 5 bp) for the 5′-end adapter. The next step was read trimming based on quality scores (mean *Q* ≥ 25), Ns (number of *N* bases lower than 10%) and poly-A tail prediction (minimum of 5 bp of A/T at both ends). This step was performed using PRINSEQ (v.0.19.5) (Schmieder and Edwards, 2011), which also filtered very small reads (length < 15 bp). An alignment against the *A. mellifera* genome (assembly version 4.5) was run using TopHat (Trapnell et al., 2009) (v.2.0.7), guided by the respective RefSeq (Release 55) transcript coordinates. The genomic alignments were then submitted to Cufflinks (Trapnell et al., 2010) (v.2.0.2) for transcript assembly, estimation of their abundances and testing for differential expression between EcR-KD and control samples. The Cufflinks procedures were also guided by the RefSeq transcript coordinates. The expression estimates were properly normalized considering ambiguous alignments, and corrected for fragment bias (Roberts et al., 2011). The Poisson fragment dispersion model was used in the comparison analysis. Cufflinks calculates the FPKM (Fragments Per Kilobase of exon per Million fragments mapped), log2-fold-change and *q*-value (*p*-value adjusted by False Discovery Rate, FDR). However, the log2-fold-change was recalculated after adding an offset of 1 to FPKM values in order to enable comparison involving samples without expression (zero) and to reduce the variability of the log ratios for low expression values (less than one). The functional annotation was done using Blast2GO (Conesa et al., 2005) (v.2.5), InterProScan (Mulder and Apweiler, 2007) (v.5-RC6), RefSeq transcript annotation and finding the Reciprocal-Best-Hit of *A. mellifera* RefSeq proteins against *D. melanogaster* proteins database (FlyBase r5.49) using blastp. The Blast2GO annotation pipeline was run based on blastp results of RefSeq proteins against nr database.

Computational processing of the Small RNA-Seq reads comprised the following steps: (i) initial sequence quality filtering based on unidentified bases; (ii) rRNA read filtering based on matches against SILVA database (Release 115); (iii) sequence adapter clipping using CutAdapt and Scythe; (iv) read trimming based on quality scores, Ns and poly-A^+^ tail prediction. All of these procedures were performed using PRINSEQ in the same way as described above. After each one of these preprocessing steps, an alignment against the *A. mellifera* genome (assembly version 4.5) was performed using the reads that had not already been aligned at each previous alignment step. Finally, all the alignment results were concatenated and transformed into a proper format to identify miRNAs. For this purpose, any splitted alignments were excluded.

Genomic alignments were performed using TopHat and the other alignments were performed using Bowtie2 (Langmead and Salzberg, 2012) (v.2.0.6). miRNA digital expression (MDE) levels were obtained by analysis with miRDeep2 (Friedländer et al., 2012) (v.2.0.0.5^*^), which provides the counts of reads mapped to the *A. mellifera* miRNA dataset in miRBase (Release 19). The original miRDeep2 code was modified to provide read counts for mature miRNAs instead of each precursor, and then the log2-fold-change was calculated and statistical significance was assessed using the method proposed by Audic and Claverie (1997) with adjustment by FDR.

## Results

### EcR-A and EcR-B transcript variant identification in honeybees

**Figure 1:**
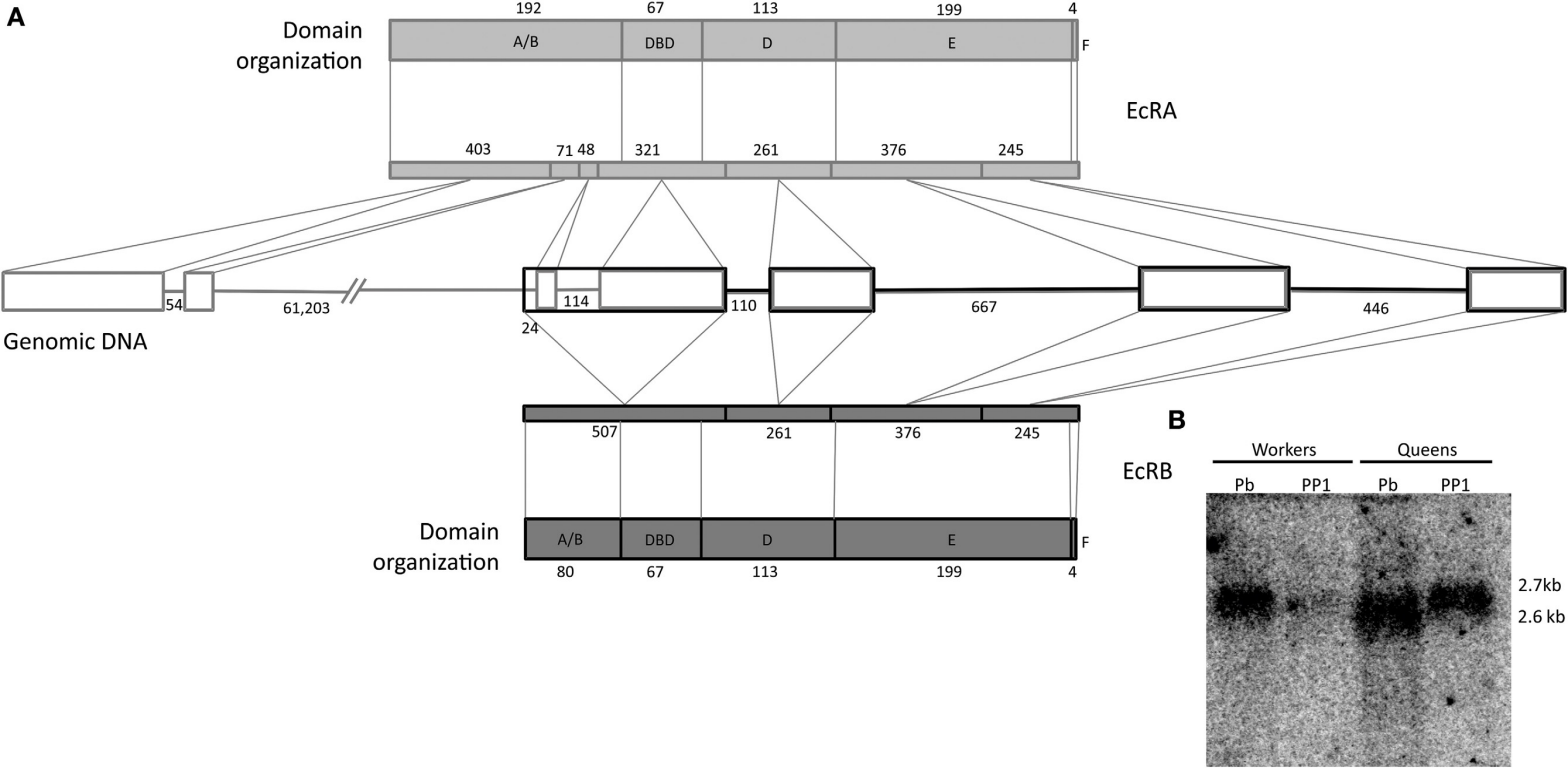
**Gene and protein organization of honeybee EcR**. **(A)** Genome: rectangles represent exons, and lines are introns, both are indicated with their respective numbers of nucleotides. Protein domains: Shown are the five domains with their respective amino acid numbers. The lenght of the coding sequence of each exon within the respective EcR domain is marked by oblique lines. 5′ and N termini are on the left. **(B)** Northern blot showing the expression of the two *A. mellifera EcR* transcript variants. Approximately 15 μg of total RNA was applied per lane. The radioactively labeled probe is a 160 bp fragment that included part of the DBD coding region. PP1, early prepupa; Pb: brown-eyed pharate-adult with unpigmented cuticle.

Two transcript variants, EcR-A (Accession numbers NM_001098215.2) and EcR-B (NM_001159355.1) of 2635 and 2782 nucleotides, respectively, have been identified for the *A. mellifera EcR* gene (Takeuchi et al., 2007 and Watanabe et al., 2010). The difference in nucleotide length was shown to reside within the 5′ end, resulting in amino acid sequence variation in the N- modulator A/B domain. Conceptual translation of the nucleotide sequences resulted in a putative EcR-A protein consisting of 629 amino acid residues and an EcR-B protein of 557 amino acids, both sharing a 452 amino acid sequence in the carboxy terminal (Figure 1A). Northern blot analysis using a C-terminal *EcR* probe showed hybridization bands of approximately 2.7 kb and 2.6 kb (Figure 1B), mainly in queen samples, but since we did not aim at quantifying, the respective band density does not necessarily represent difference in transcript levels between the two castes. Nonetheless, this result reveals that the two transcripts indeed have small differences in length, this supporting the *in silico* evidence.

### Developmental profiles of the EcR transcript variants A and B

**Figure 2:**
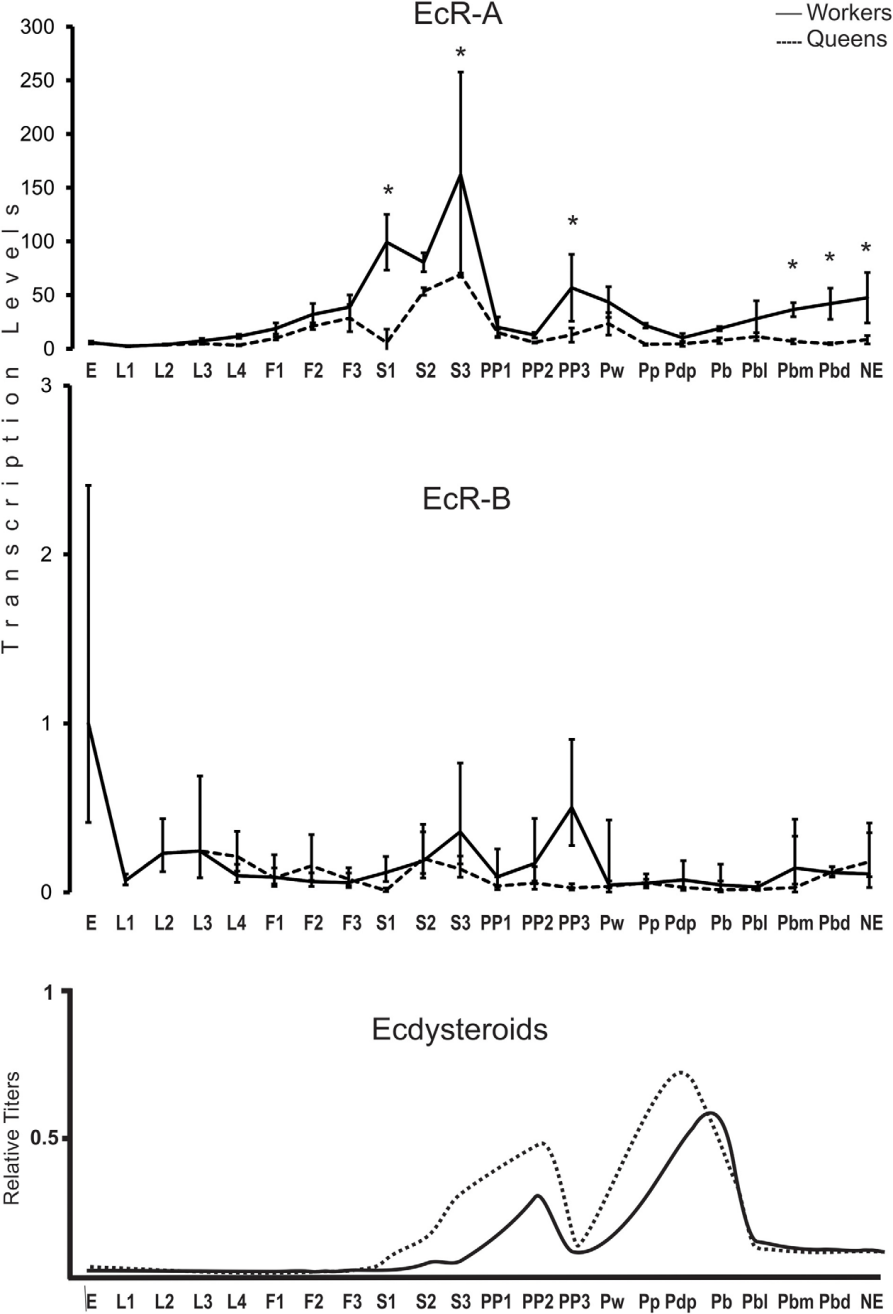
**Developmental expression profile of the *EcR* gene in *A. mellifera* queens and workers**. EcR-A and EcR-B transcript levels were measured by RT-qPCR. Bars represent means ± S.E.M. of three biological replicates, each run as three technical replicates. Asterisks indicate a statistical difference (Mann–Whitney Test, *P* ≤ 0.05) between queens and workers for the respective developmental phase. See Table 1 for a description of the developmental phases. Hormone titers are based on Rachinsky et al. (1990) and Pinto et al. (2002).

Using variant-specific primers we quantified the transcript levels of *EcR-A* and *EcR-B* covering the entire postembryonic development for honeybee queens and workers (Figure 2). Three major findings are worthy of note: (i) transcripts representing the *EcR-B* variant are predominant in embryos (Mann–Whitney Test, *P* ≤ 0.05), but these transcript levels decline at the transition to the first larval instar, and it is the *EcR-A* variant which is then predominantly expressed during the end of the larval stage (fifth instar) and pupal stage; (ii) at several time-points, *EcR* expression is higher in workers than in queens (Mann–Whitney Test, *P* ≤ 0.05); and (iii) there is a clear discrepancy between circulating ecdysteroid levels and the developmental expression of EcR-A.

Major caste differences in EcR-A expression were seen to accompany the larval/pupal metamorphic molt. As soon as the larvae were no longer fed by nurse bees and the brood cells were closed, the EcR-A levels were increased by two orders of magnitude in cocoon-spinning worker larvae (S1–S3 phases). A rise was also seen in EcR-A levels in cocoon spinning queen larvae, but this was significantly lower than in workers (Mann–Whitney Test, *P* ≤ 0.05). A similar pattern was also seen for the EcR-B variant, but at much lower modulation. Interestingly, the EcR expression levels were then decreased for both variants and in both castes at the onset of the prepupal development (PP1), marked by the appearance of apolysis fluid separating the fifth instar larval cuticle from the newly synthesized pupal cuticle in the head region. A new rise in the transcript levels of both variants was then seen at the end of the prepupal development (PP3), but this was primarily evident in workers (Mann–Whitney Test, *P* ≤ 0.05). EcR-A and EcR-B transcript variants remained at low levels during the pupal and early pharate-adult stages (Pw to Pbl phases) before they showed another steady increase, but again mainly so in workers (Mann–Whitney Test, *P* ≤ 0.05).

### Transcriptional response of EcR to artificially augmented ecdysteroid and JH titers

**Figure 3:**
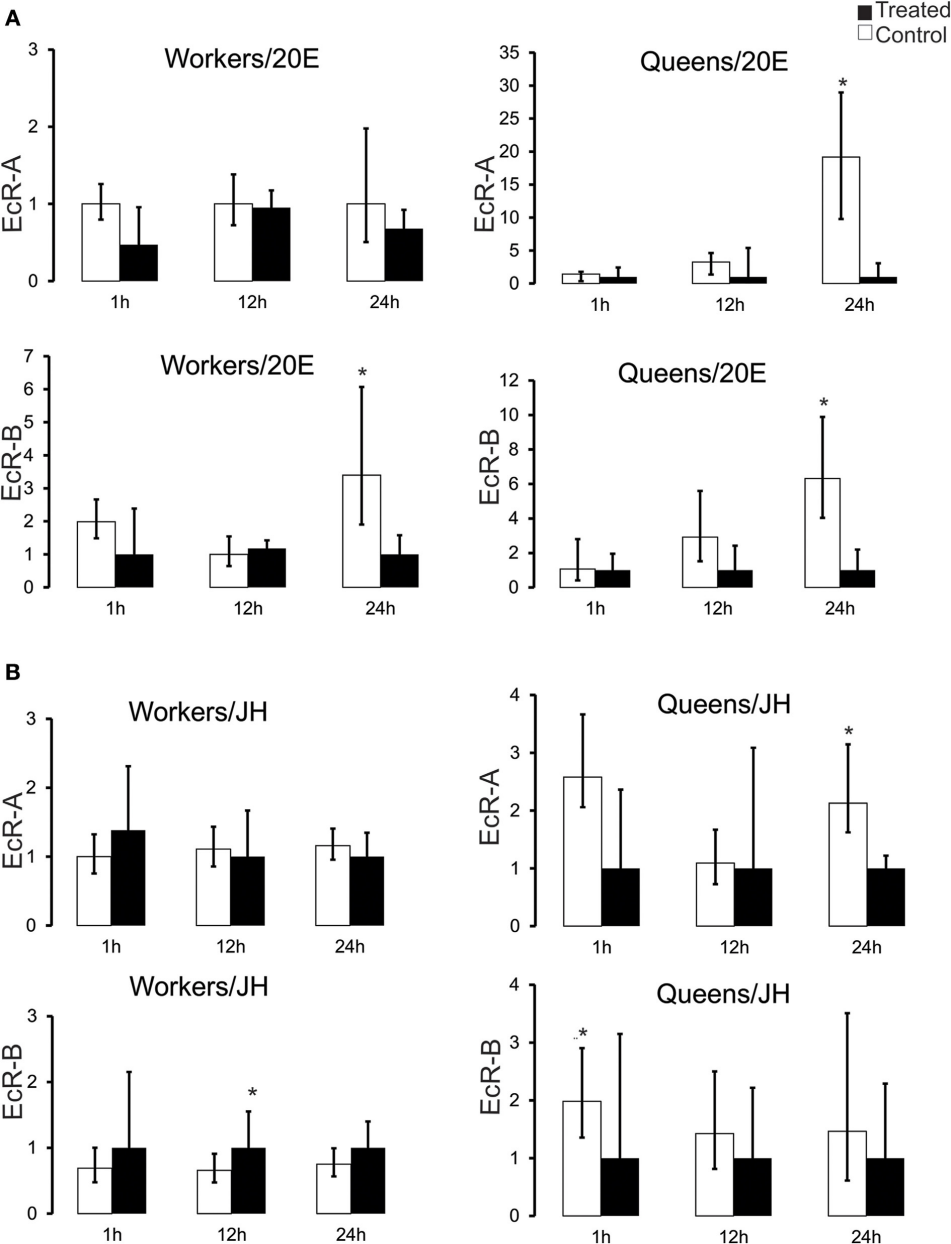
***EcR* expression response to experimentally augmented levels of (A) 20-hydroxyedysone (20E) and (B) juvenile hormone (JH) in honeybee castes**. Aliquots of 5 μg of 20E or 10 μg of JH-III were applied to brown-eyed pharate-adults with unpigmented cuticle (Pb phase). RNA samples from fat bodies were obtained after 1, 12, and 24 h after hormone applications. Bars represent means ± S.E.M. of three samples. Each biological replicate consisted of 3–7 Pb-phase workers and each was run as three technical replicates. Statistical differences (^*^P = 0.05) in gene expression between treated and control groups were assessed by Mann–Whitney Test.

So as to better understand the relationship between hemolymph hormone titers and hormone receptor expression, especially the remarkable divergence in the pupal stage, we treated Pb-phase workers and queens, as these are at the transition from pupal development *per se* to the pharate adult stage, with JH and 20E. At the Pb-phase the ecdysteroid titer is rapidly declining in both castes after having gone through the maximum peak at the preceding Pp phase (Pinto et al., 2002), while JH levels are still basal (Rembold, 1987). The transcriptional responses for the two EcR variants assayed by RT-qPCR revealed a general repressive effect of both hormones at 24 h after application (Figure 3). In queens, 20E injection elicited a repressive effect on both EcR variants. Mean transcript levels were diminished at 12 h after 20E injection and were significantly lower at 24 h (Mann–Whitney Test, *P* ≤ 0.05). In workers this was the case only for the *EcR-B* transcript and only at 24 h (Figure 3A).

The effect of exogenous JH on *EcR* expression was not as clear-cut as that elicited by 20E treatment. While there was no apparent effect on *EcR-A* transcripts in workers, the *EcR-B* levels showed slightly elevated means at all time points (Figure 3B), and these were significantly higher at 12 h following hormone treatment (Mann–Whitney Test, *P* ≤ 0.05). Interestingly, in the queen caste the response to JH treatment appeared to be opposite to that seen in workers, with mean EcR-A and EcR-B transcript levels diminished already at 1 h after treatment and significant differences apparent at 1 h in the case of *EcR-B* and at 24 h for *EcR-A* (Mann–Whitney Test, *P* ≤ 0.05). These results indicate a repressor effect of high circulating ecdysteroid levels on *EcR* expression in both castes and a differential response to JH, with workers responding positively and queens negatively to elevated JH levels.

### EcR knockdown in pharate-adult honeybee workers significantly downregulates the expression of candidate target genes

So as to understand the role of the *EcR* gene in honeybee development, beyond the correlation analysis between transcript levels of the two EcR variants and hormone levels, we experimentally decreased the *EcR* gene functionality by an RNA interference approach. We herein focused on the *EcR* response in workers during the pharate-adult to adult transition because only one of the two transcript variants, *viz*. *EcR-A*, undergoes a gradual increase at this developmental interval, and only so in the worker caste (Figure 2). We expected this to give not only more clear-cut results and insights into the role of the predominant EcR variant, but also into still very little understood aspects of morphogenetic processes taking place in developing adult honeybees.

**Figure 4:**
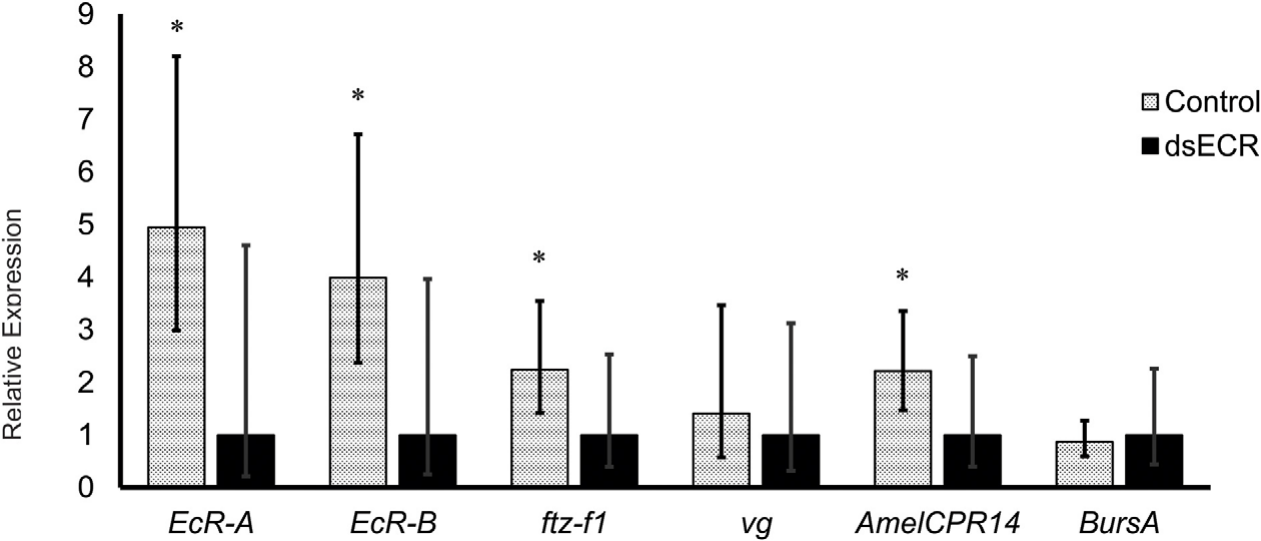
**Relative transcript levels of four candidate EcR-target genes following *EcR* knockdown in honeybee workers**. Twelve pharate-adult workers (Pbl phase) were injected with 5 μg of EcR-dsRNA (KD) or GFP-dsRNA (C, control) and sampled just after adult eclosion. Bars represent means and S.E.M of 12 biological replicates, each run as three technical replicates. ^*^Indicates a statistically significant difference (Mann–Whitney Test, *P* ≤ 0.05).

The dsRNA fragment used in this experiment represented an EcR region shared by the two transcript variants and its injection resulted in a reduction of 79.8 and 74.9% for *EcR-A* and *EcR-B* mRNA levels, respectively (*P* < 0.001, Student's *t*-test; see Figure 4). A mortality of 10% was observed in both EcR- (KD) and GFP-dsRNA treated (control) bees. A proportion of dsRNA-injected bees showed alterations in cuticle pigmentation and wing development, similar to previously reported observations by Barchuk et al. (2008) when studying *ultraspiracle* function. Based on the strong knockdown response we next assayed the transcriptional response of four candidate target genes, these being a homolog of the *D. melanogaster ftz-f1* gene, the *vg* gene, and two genes involved in adult cuticle formation (*AmelCPR14* and *BursA*). The *ftz-f1* gene was included in this analysis because in *D. melanogaster* it acts as a competence factor for the response to 20E; furthermore, EcR also inhibits *ftz-f1* expression in *D. melanogaster* mid-prepupa, thus temporarily impairing the larval-to-pupal transition in response to the second 20E peak (King-Jones and Thummel, 2005). In pharate-adult honeybees, the levels of *ftz-f1* transcripts were seen to increase (data not shown) concomitantly with the levels of *EcR-A*, suggesting a synergistic action of the two genes. In addition, the increase in the levels of the two genes coincides with the increase in the expression of genes encoding enzymes and proteins needed for the complete differentiation of the adult cuticle (Soares et al., 2007, 2011, 2013; Elias-Neto et al., 2010). Similarly, in *D. melanogaster* the expression of *ftz-f1* has recently been related to adult cuticle formation and eclosion (Sultan et al., 2014). The analysis of *ftz-f1* transcript levels in newly emerged workers (*N* = 12), i.e., approximately 2 days after injecting dsRNAs, showed that *ftz-f1* expression was significantly decreased in EcR-KD bees (*P* ≤ 0.05, Student's *t*-test) (Figure 4). A significant effect of the EcR knockdown was also seen for the cuticular protein gene *AmelCPR14*, but not for the *Burs A* gene that encodes a subunit of the neurohormone Bursicon. The significant reduction in the expression of a cuticular protein gene following EcR-RNAi is consistent with the ecdysteroid-related expression of these genes in developing honeybees (Soares et al., 2007, 2011, 2013). An interesting though not easily explained finding was that *vg* gene expression was not significantly affected by reducing EcR function, although the mean *vg* transcript levels were slightly reduced compared to the non-target dsRNA control. This was surprising as *vg* gene expression has been shown to gradually increase in pharate-adult honeybee females, and this increase was thought to be related to ecdysone levels (Barchuk et al., 2002; Piulachs et al., 2003).

### EcR knockdown affects the poly-A^+^ profile of newly emerged workers

So as to understand EcR functions during the pharate-adult to adult transition of honeybee workers on a more global scale we compared the poly-A^+^ transcriptomes of EcR-KD and GFP-injected (control) bees. After filtering of the raw data we obtained 112,659,148 reads for the KD and 71,050,536 reads for the control samples. Most of these reads were 50 nt long. This data has been submitted to the Sequence Read Archive (SRA, NCBI, http://www.ncbi.nlm.nih.gov/sra) under the Accession Number SRX700299. As we had only one RNA sample set per group (two libraries, no replicates), the estimate obtained by Cuffdiff analysis was that 234 loci were differentially expressed [absolute log2 (fold change) >1; *q*-value = 0.05; FPKM > 5 in at least one library] (Table S2). Among these, 121 code for known protein products, and 100 of these were upregulated in KD pharate-adults and 21 were downregulated (overexpressed in control bees; Table 2). The five times higher number of differentially expressed genes in the EcR-KD group indicates that during the pharate-adult to adult transition more genes may be repressed by ecdysone than are induced.

**Table 2:**
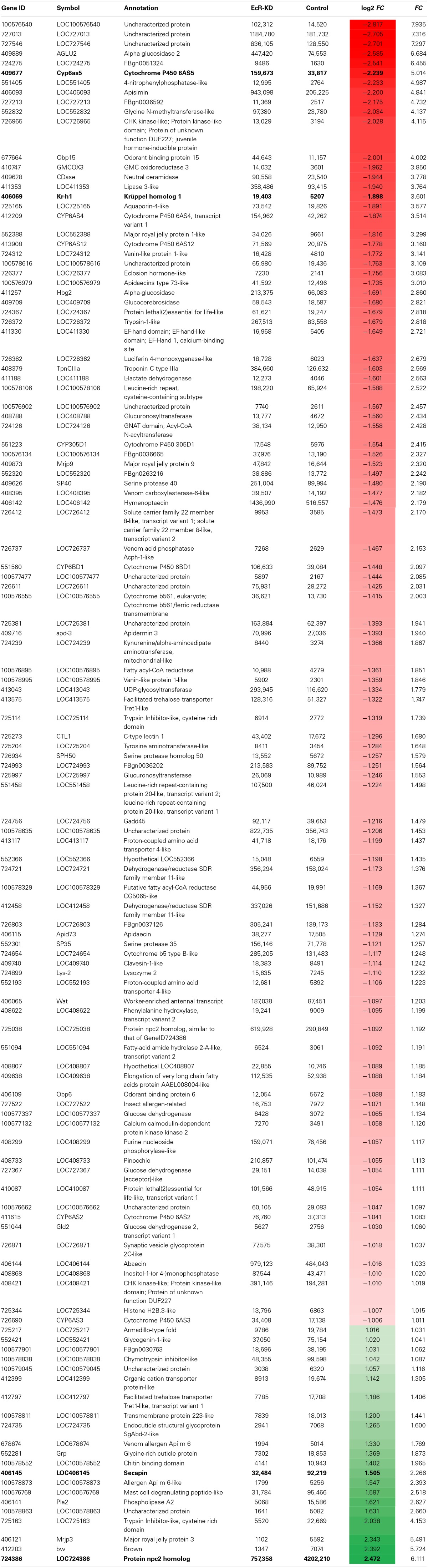
**Protein-coding genes (121) that were differentially expressed in EcR knockdown (EcR-KD) bees (*FC* ≥ 2; *q*-value ≤ 0.0015)**.

*Negative values of log2 FC indicate genes that are upregulated in EcR-KD bees (highlighted in red); positive values indicate downregulation in EcR-KD bees (highlighted in green). Genes shown in bold had their transcription patterns validated by qPCR. Expression values measured as FPKM (Fragments Per Kilobase of exon per Million fragments mapped). This list contains only the genes with FPKM values of 5 for any of the two samples*.

In terms of functional assignments the following conclusions can be drawn. Seven genes among the ones upregulated in the KD group code for cytochrome P450 proteins (Table 2 and Figure S1A), six of these belonging to CYP clade 3 (CYP6AS2, CYP6AS3, CYP6AS4, CYP6AS5, CYP6AS12, and CYP6BD1) and one (CYP305D1) to CYP clade 2 [for clade assignments of honeybee cytochrome P450 genes see (Claudianos et al., 2006)]. A second protein family that was well-represented among the upregulated genes in the KD group is that encoding Major Royal Jelly Proteins (MRJP1 and MRJP9) and an MRJP-associated protein, apisimin. A third class is represented by hormone response-related genes: a gene encoding a JH-inducible protein, a gene encoding a honeybee eclosion hormone (EH) homolog, and *krüppel-homolog* 1, an immediate response gene regulated by the JH receptor (Bellés and Santos, 2014). Nonetheless, the genes with the highest differential expression index are three genes encoding transcripts of unknown function and without conserved domain evidence (LOC100576540, LOC727013, and LOC727546). The fourth highest upregulated gene in the KD group encodes an α-glucosidase, an enzyme that converts the disaccharide sucrose into glucose and fructose and is, thus, critically involved in carbohydrate metabolism. Another three genes in the top gene list are also related to metabolic functions, these being transcripts for a glycine N-methyltransferase-like, a glycine-methanol-choline (GMC) oxidoreductase 3 and a lipase 3-like protein. Furthermore, three genes upregulated in KD bees, the GMC oxidoreductase 3, a UDP-glycosyltransferase (LOC 413043) and a glucuronosyltransferase (LOC 725997), could be related to ecdysteroid metabolism and function.

The genes downregulated in EcR-KD bees are listed at the bottom of Table 2. They are represented with positive fold change values, as these were calculated as relative to the control group. In contrast to the upregulated genes, those that were downregulated are not as clearly associated to putative functions during the pharate-adult to adult transition, except for the LOC724735 and *Grp* genes that encode structural cuticle proteins needed for the construction of the adult cuticle at this stage. The gene with the highest overexpression index in the control group codes for a Niemann-Pick type protein (NPC2), that is, genes involved in cholesterol metabolism-related syndromes and diseases (another *npc2*-type gene was found slightly overexpressed in the KD bees). Next are three transcripts possibly related to venom gland function, encoding a phospholipase, secapin and a putative mast cell degranulating peptide (Table 2 and Figure S1A). Also downregulated was the *brown* gene, which encodes an ABC-2 type transporter protein, and a gene coding for a Major Royal Jelly Protein (MRJP3).

A more global analysis on the entire set of differentially expressed genes was done based on Gene Ontology (Blast2GO and InterProScan) using Fisher and Kolmogorov–Smirnov statistics. This confirmed that the poly-A^+^ RNAs representing genes upregulated in the KD group are enriched in proteins participating in metabolic pathways, particularly ones with catalytic and oxidoreductase activities (Table S3).

So as to validate the poly-A^+^ RNA-sequencing results we then chose two genes revealed as upregulated in the KD group (*Cyp6as5*, a P450 protein coding gene, and *kr-h1*, a gene encoding the JH response factor Krüppel homolog-1) and two downregulated genes (LOC406145, *secp* and LOC724386, *npc2*). For these we designed or selected from the literature gene-specific primers and ran RT-qPCR assays. The expression pattern was confirmed for all four genes (Figure S1A), thus providing further evidence that the 234 poly-A^+^ RNA coding genes found as differentially expressed between treated and control bees are under EcR control.

### EcR knockdown affects the miRNA profile of newly emerged workers

We obtained a total of 31,171,886 and 33,683,147 reads of small RNAs from the KD and control sequence libraries, respectively. This data has been submitted to the Sequence Read Archive (SRA, NCBI, http://www.ncbi.nlm.nih.gov/sra) under the Accession Number SRX700299. After filtering the raw data, we focused on the discovery of miRNAs linked to the EcR network. A total of 4,436,511 reads of the KD samples (~13.2%) and 10,557,117 reads of the control sample (~33.9%) mapped to known honeybee mature miRNAs (available in miRBase version 19), suggesting that EcR disruption causes a general downregulation of miRNA families. We considered as “expressed” those miRNAs with more than 10 reads represented in at least one library. By doing so we retrieved a total of 132 known miRNAs expressed in newly emerged workers, most of them (124) in both conditions (Table S4). In order to find a set of miRNAs whose transcription is significantly affected by the EcR pathway, we filtered the Cuffdiff results by selecting miRNAs with expression differences higher than 1.2-fold and a *q*-value < 0.05 between KD and control bees. We found 60 downregulated and 10 upregulated miRNAs in KD samples compared to controls (Table 3). These data were then further validated by RT-qPCR assays for the following miRNAs: miR-14, miR-100, miR-125, miR-133, miR-375, miR-3728, and miR-3771 (Figure S1B).

**Table 3:**
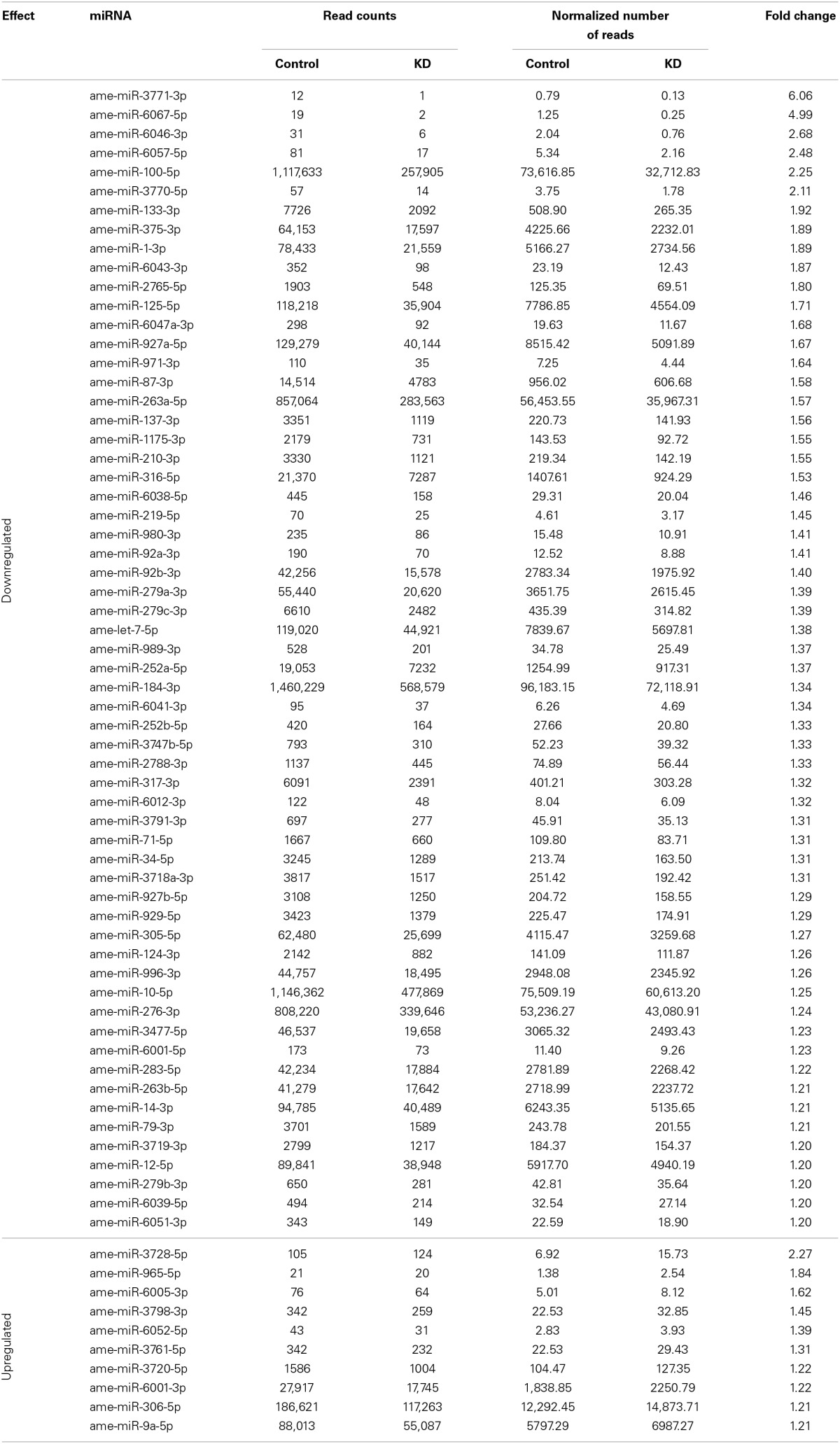
**miRNAs that were differentially expressed in EcR-knockdown bees**.

*Only miRNAs with Fold-change >1.2 are listed*.

## Discussion

### The honeybee EcR transcript variants and their developmental regulation

The existence of more than one EcR isoform is commonplace in insects, including the honeybee, for which two transcript variants, *EcR-A* and *EcR-B* had been found (Takeuchi et al., 2007). First shown for *D*. *melanogaster* (Talbot et al., 1993) and then for the red flour beetle *Tenebrio molitor* (Mouillet et al., 1997), the extensive review of insect EcR isoforms by Watanabe et al. (2010) showed a high similarity in their nucleotide and amino acid sequences in most of their functional domains, except for the N-terminal region including the variable A/B modulator domain, which might allow for the recruitment of different co-activators/co-repressors (Tora et al., 1988; Kato et al., 1995; Watanabe et al., 2010).

First, we confirmed by northern blotting the expression of the two *EcR* variants in honeybee queens and workers. Then, we compared their temporal expression profiles to the hemolymph ecdysteroid titers of fifth instar queen and worker larvae (F1-PP3 phases) (Rachinsky et al., 1990). The results for the developmental expression profiles of the two ecdysteroid receptor variants are surprising in two aspects. First, contrasting with the hormone titers, which are higher in queens than in workers, the *EcR* transcript levels were found to be higher in workers, especially so for *EcR-A*. Second, there was a marked drop in *EcR* expression at the beginning of the prepupal phase (PP1), i.e., exactly when the hemolymph ecdysteroid levels increase to reach a developmental peak at the subsequent PP2 phase. Strikingly as well, the transcript levels for both EcR variants remained at low or basal levels during the pupal and early pharate-adult stages (Pw to Pbl phases), even though the ecdysteroid hemolymph titers are at a maximum during this period (Feldlaufer et al., 1985; Pinto et al., 2002).

The switch from EcR-B expression in the embryonic stage to EcR-A as the predominant isoform in the fifth larval instar and pupal stage reflects a change in the processing of an eventual long pre-mRNA, or a shift in transcription start site utilization (our RNA-Seq data are in support of the latter possibility and even suggest the existence of a third EcR transcript variant). Since the *EcR* gene is known to be induced after an ecdysteroid pulse (Karim and Thummel, 1992; Davis and Li, 2013), the production of *EcR-B* mRNA in honeybee embryos would require the presence of steroid hormones, which is indeed the case. Makisterone A, the predominant ecdysteroid in *A. mellifera* (Feldlaufer et al., 1985), has been shown to be present in ovaries in quite large amounts (Feldlaufer et al., 1986a), and unpublished data from our laboratory also confirm the presence of ecdysteroids in developing embryos. High levels of ecdysteroids in ovaries have also been shown for bumblebee queens (Geva et al., 2005) and queens of a swarm-founding neotropical wasp, *Polybia micans* (Kelstrup et al., 2014). Embryonic ecdysteroids can be synthesized by enzymatic conversion from inactive conjugates stored during oogenesis (Dorn, 2000) or, as seen in mosquitoes, transferred by males during copulation (Baldini et al., 2013).

Since makisterone A is the predominant ecdysteroid compound in queen ovaries (Feldlaufer et al., 1986a) and also in pupal-stage hemolymph (Feldlaufer et al., 1986b) and 20E is not negligible in prepupal hemolymph (Rachinsky et al., 1990), the observed embryonic-to-larval EcR isoform switch may be linked to variation in the ecdysteroid composition circulating in the hemolymph, throughout a bee's life cycle. This could not only be responsible for the observed differential *EcR* transcription, but also for the formation of different hormone/receptor complexes with potentially different target genes thus, governing separate physiological processes. 20E, for example, might have retained a role in reproductive physiology, as suggested by Takeuchi et al. (2007), whereas makisterone A may have been co-opted for governing postembryonic development, as suggested for *Dysdercus fasciatus* (Feldlaufer et al., 1991). Nonetheless, for honeybees such “division of labor” in ecdysteroid compounds is still highly speculative, especially since the ecdysteroid hemolymph levels in adult honeybee queens and workers are continuously low, this making it rather unlikely that these steroid hormones may play a major role in the reproductive female physiology (Hartfelder et al., 2002). Instead, they seem to be preferentially stored in the developing follicles.

The second and third major findings mentioned above are that the *EcR-A* transcript levels are higher in worker than in queen development, and that there is no positive, but rather an apparently negative correlation between hormone levels and hormone receptor transcript levels. This stands in stark contrast to the developmental pattern of the hemolymph ecdysteroid titers in the two castes, which are higher in queens than in workers, particularly so during larval-pupal metamorphosis (Rachinsky et al., 1990). The ecdysteroid titer in last instar queen larva rises as soon as the brood cells are closed and the larvae start to spin their cocoons (S1-stage), while in workers this was only seen in the late spinning (S3) to early prepupal (PP1) phases. Furthermore, the peak in edysteroid levels reached during the prepupal phase (PP2) is twice as high in queens compared to workers (Rachinsky et al., 1990). The negative correlation between ecdysteroid levels and *EcR* expression is particularly evident at two time points: in prepupae, when ecdysteroid levels are high in the PP1–PP2 phases, just as the EcR-A expression pattern undergoes a valley, and in the pupal stage, when the ecdysteroid levels are high in both castes at the Pp phase, before dropping in the Pb-Pbl phases (Pinto et al., 2002). It is only after this drop in circulating hormone levels that *EcR-A* transcription is resumed, particularly so in the worker caste, and strikingly, it is during the subsequent Pbm and Pbd phases that the ecdysteroid levels are again lower in workers than in queens (Pinto et al., 2002).

This apparent negative correlation between hormone and hormone receptor levels was suggestive of a repressive action of high concentrations of circulating ecdysteroids on the expression of their receptor gene. To test this we manipulated the endogenous hormone levels by treating Pb pharate-adults with either 20E or JH. The results of the 20E injection experiments showed that a prolonged and excessive presence of ecdysteroids had a repressive effect on EcR-A and EcR-B expression, especially so in queens (Figure 3A). Interestingly, workers seem to be more resilient to this repressor effect, as there was no significant reduction in EcR-A transcript levels, comparable to that seen in queens, or for EcR-B in both castes. Such resilience was also denoted in the JH application experiment, where EcR-A transcript levels in workers remained little affected compared to those in queens and for EcR-B in both castes 24 h after the 20E injection. Strikingly, JH appeared to have opposite effects on EcR-B expression in the two female castes, showing a positive effect in workers and a negative one in queens. These differences of hormone effects on *EcR*-expression related to caste certainly deserve a closer look in future experiments.

Repressive effects of high concentrations of ecdysteroids on EcR-expression are, however, not new and are likely to be a general feature of hormone systems that underly cyclical events in morphogenesis and physiology. For instance, similar results were described for *Manduca sexta*, where low concentrations of 20E induced *EcR* expression while high concentrations repressed the expression of this gene (Jindra et al., 1996). Like ours, these results suggest that the *EcR* gene responds positively to a slight increase in ecdysteroids, whereas high hormone levels are repressive. In fact, as we could see, *EcR* expression actually appears to precede the rise in hormone levels, for instance in the S1–S3 phases, when the circulating ecdysteroid levels start increasing (Rachinsky et al., 1990), but EcR-A and also EcR-B transcript levels have already undergone a steep rise. A repetition of this pattern can be inferred for the pupal ecdysis event, occurring between PP3 and Pw, when the ecdysteroid titers undergo a sharp drop, but EcR-A and EcR-B are on the rise (mainly in workers), and drop once the ecdysteroid titers build up to maximal values in the Pp phase. It is such cyclical events, the molts, that are synchronized by the ecdysone/ecdysone receptor complex action, and this is primarily seen in the epidermis, the main organ of cuticle synthesis. In the honeybee, several cuticle protein genes were shown to be regulated by ecdysteroids (Soares et al., 2007, 2011, 2013; Elias-Neto et al., 2010).

### RNAi-mediated knockdown reveals EcR regulated genes in developing adults

Upon comparing the sequencing results of the poly-A^+^ libraries for EcR knockdown (EcR-KD) and control groups, the Cutdiff analysis classified 234 loci as differentially expressed. Among these, 121 were annotated as coding for known protein products or, from another point of view, 113, i.e., one half, represent loci for unknown, not annotated products, which could be either proteins or long non-coding RNAs. Especially the latter are still “dark matter” in the honeybee genome, represented by many ESTs in the databases, but only four long non-coding RNAs are so far characterized to some detail (Sawata et al., 2002; Humann et al., 2013).

Among the genes with known orthologs or sequence similarity in functional domains, 100 were overexpressed (fold change > 1) in the EcR-KD group and 21 in the control group, this indicating that apparently more genes are repressed by the ecdysone/EcR receptor complex than are activated. Furthermore, a Gene Ontology and KEGG pathway analysis showed that there is little overlap in gene functions between the two sets of differentially expressed genes (DEGs). As mentioned above, cytochrome P450 genes are strongly represented among the DEGs. While cytochrome P450 genes are a large gene family, strongly related to detoxification processes, this family has undergone considerable reduction in honeybee genome evolution (Claudianos et al., 2006). This reduction is, however, denoted only in certain clades of the P450 enzymes, but not in the clades comprising the genes found in our EcR-RNAi experiment. Unfortunately, there is no further functional or tissue/cell type information available for the five cytochrome P450 genes, especially whether or not they may be related to steroid synthesis or metabolism. Nonetheless, similar findings as the ones we report here were also denoted by Davis and Li (2013) in their genomic screen for ecdysone and EcR-dependent gene expression in *D. melanogaster*.

A second group of overrepresented genes that called attention was the hormone response-related genes, as these may provide a link between JH and ecdysteroid action during the pharate-adult to adult transition in honeybees. For this group we found three genes as overexpressed in the EcR-KD group, *viz*. a JH-induced protein, *kr-h1*, and an Eclosion hormone-like (*EH-like*) gene. *kr-h1* is certainly the most interesting gene in this set, as it represents a direct readout of the activity of the JH response in target tissues (Lozano and Belles, 2011; Bellés and Santos, 2014). As *kr-h1* has previously been identified in a screen for ecdysone-response genes in *D. melanogaster* (Beckstead et al., 2005), the current identification of this gene in the EcR-KD group provides experimental evidence toward a mechanistic explanation for the modulation of vitellogenin induction in honeybee pharate-adults, where *vg* expression is caste-specifically induced by JH and counteracted by ecdysteroids (Barchuk et al., 2002). Overexpression of an *EH-like* gene in the EcR-KD group was not unexpected, as EH is synthesized in response to declining ecdysteroid titers and is part of the ecdysis triggering signaling cascade (Zitnan and Adams, 2012). Interestingly, other three upregulated genes in EcR-KD bees may have roles in ecdysteroid metabolism and function. Several GMC oxidoreductase genes in diverse insects, including *A. mellifera*, are clustered in an evolutionary conserved tandem array with potential to be co-regulated for a common function related to ecdysteroid metabolism (Iida et al., 2007). The products of LOC413043 and LOC725997 may regulate ecdysteroid titer and function since the enzymes encoded by these genes catalyze the transfer of glucose from UDP-glucose to ecdysteroids, and thus are possibly related to ecdysteroid inactivation (O'Reilly and Miller, 1989).

Overexpression of members of the Major Royal Jelly Protein (MRJP) family can be interpreted in the context of a repressive action of the ecdysone/EcR receptor complex on genes of the adult honeybee life cycle (the only exception being the gene coding for the MRJP3, which was overexpressed in control bees). The *mrjp* gene family with its nine members is a lineage-specific extension in the genus *Apis*, from a single *mrjp-like* gene within the *yellow* genes complex (Drapeau et al., 2006). Even though these proteins are highly expressed in the hypopharyngeal glands of nurse worker bees, constituting the major protein fraction of the glandular secretions fed to larvae (royal jelly and worker jelly), expression of the *mrjp* genes is neither exclusive to this tissue nor is it restricted to the worker caste. Especially *mrjp9* has been shown to be broadly expressed, in different tissues of adult workers and also in queens and even drones (Buttstedt et al., 2013). In contrast to *mrjp9*, *mrjp1* expression is more tissue-specific, being highest in heads (*viz*. hypopharyngeal glands) of nurse bees, with expression levels being considerably lower in other body parts, castes and sexes (Buttstedt et al., 2013). MRJP1 is the predominant MRJP moiety in royal jelly, present as oligomers of MRJP1 subunits, which are held together by apisimin, a small 5 kDa protein (Tamura et al., 2009). ESTs corresponding to *apisimin* were found as overrepresented in the EcR-KDS group, indicating that its expression is co-regulated with that of *mrjp1*. But this co-regulation is not due to genomic proximity, as the *mrjp/yellow* gene cluster maps to chromosome 11, whereas *apisimin* is located in chromosome 6. Interestingly, an MRJP1 monomer, royalactin, was found to be an important factor in caste development, acting through the Egfr signaling pathway (Kamakura, 2011).

Among the EcR-KD group genes we also identified *obp15*, which encodes a putative odorant binding protein. Some *obp* genes were also found to be under negative EcR control in *D. melanogaster*, including the *obp15* and *obp6* genes (Davis and Li, 2013). Forêt and Maleszka (2006) had previously shown that *obp15* is expressed in the antennae of adult bees and also in young larvae but not in pupae. The high ecdysteroid levels in honeybee hemolymph during the pupal to pharate-adult transition, thus, appear to repress *obp15* expression, and possibly also other members of this complex gene family.

Among the genes overrepresented in the transcriptome of the control group (downregulated in EcR-KD bees), the first in the top ten list is annotated as *npc2*. Genes of this family are associated with Niemann-Pick syndromes and diseases affecting cholesterol metabolism (Carstea et al., 1997). In *D. melanogaster*, *NPC* mutations cause intracellular enrichment of cholesterol, reduced ecdysteroidogenesis and death in the first larval instar. The fact that this condition could be fully rescued when an excess of dietary cholesterol was given to these mutants indicated that the ecdysone biosynthesis pathway is intact, but precursor processing is not (Huang et al., 2007). Interestingly, in honeybees, as in other insects, the major ecdysteroid is not ecdysone or its derivative 20E, but makisterone A, an ecdysteroid methylated at C24 (Feldlaufer et al., 1985, 1986a,b; Rachinsky et al., 1990), possibly due to a lack or restriction in C24-demethylation of a phytosterol precursor. The expression of two other genes overrepresented in the transcriptome of the control group has been shown to be dependent on the ecdysteroid titer. The protein encoded by LOC724735, an endocuticle structural protein (Márcia M. G. Bitondi, unpublished results) and also the *Grp* gene, renamed as *tweedle1* (*AmelTwdl1*) (Soares et al., 2011), were induced in the integument by the ecdysteroid pulse that promotes the pupal to pharate-adult transition. Thus, the functionality of the ecdysone/EcR complex is necessary for the activation of these genes. Interestingly, *mrjp3*, the third among the genes overrepresented in control bees (and thus induced by the EcR pathway), encodes one of the main MRJPs produced by nurse bees (Buttstedt et al., 2013). The *mrjp3* gene thus seems to be highly expressed by the time of adult emergence and the first days of adult life. Unlike the *mrjp1* and *mrjp2* genes, *mrjp3* reaches negligible expression levels in foragers, which, together with its distinctive amino acid sequence (Drapeau et al., 2006), supports the notion of its main function as food protein supplier to larvae by nurse bees. However, the fact that *mrjp1*, another MRJP gene highly expressed in nurse bees, was found to be repressed by the ecdysteroid pathway (see above), suggests the *mrjp3* gene is regulated in a distinct mode from the other *mrjp* genes.

### miRNAs as actors in the EcR regulatory network

Here we demonstrate that the RNAi-mediated knockdown of *EcR* function perturbs the expression of 70 miRNAs (~1/3 of the honeybee miRNAs known to date). Most of these (60) were downregulated and 10 were upregulated and we assume that these down and upregulated miRNAs are “induced” or “repressed,” respectively, by the EcR pathway as bees undergo the pharate-adult to adult transition.

Among the miRNAs that showed significant changes in abundance following *EcR* knockdown, most had already been identified in a large-scale sequencing project (Chen et al., 2010), but had no function(s) associated. Our data now lead to infer that these miRNAs are, at least, closely associated with EcR action and, consequently, connected to pupal-adult metamorphosis. In addition to these miRNAs of yet unclear functions, we also found conserved and functionally well-defined miRNAs, such as let-7, miR-1, miR-133, miR-375, miR-184, and miR-34. For example, miR-133 and miR-1 are both clustered in the mouse and fly genomes, and they play important roles in muscle development and differentiation in vertebrates and invertebrates (Sokol and Ambros, 2005; Chen et al., 2006; Boutz et al., 2007). In the honeybee, however, we found these two miRNAs to be located far apart from one another on chromosome 16. Nonetheless, they still seem to be linked in their cooperative functions, such as formation and physiology of flight muscle tissue. miR-133 has also been implicated in dopamine production (Yang et al., 2014), and high levels of dopamine were shown to coincide with rapid growth and compartmentalization of the antennal lobe neuropil, suggesting a role in the developing brain of the honeybee (Kirchhof et al., 1999). Furthermore, dopamine-derivatives are substrates for oxidation by laccases (Andersen, 2010) that are involved in tanning of the developing adult cuticle (Elias-Neto et al., 2010). Members of the *D. melanogaster let-7-C* locus (a cluster containing the *let-7, miR-100*, and *miR-125* genes) are also found in the honeybee genome. In *D. melanogaster* they are expressed in neuromusculature development of pupae and adults, and knockout flies showed disturbances in flight, reproduction and locomotion (Sokol et al., 2008). Moreover, ecdysteroid signaling was shown to be linked to the expression levels of the let-7-C cluster genes, as well as of miR-14 and miR-34 during insect development (for review see Kucherenko and Shcherbata, 2013).

Many of the miRNAs affected by *EcR* knockdown in honeybees (let-7, miR-1, miR-9a, miR-12, miR-14, miR-34, miR-79, miR-92b, miR-124, miR-184, miR-210, miR-219, miR-263a, miR-276, miR-279, miR-283, miR-305, miR-306, miR-316, miR-317) have previously been reported as putatively involved in the regulation of *D. melanogaster* immune genes, particularly those belonging to the JNK, Imd and Toll signaling pathways (Fullaondo and Lee, 2012). Accordingly, ecdysone and the ecdysone receptor complex (EcR/USP) are considered critical for innate cellular immunity (Flatt et al., 2008; Regan et al., 2013). Among these miRNAs, miR-184 is highly and/or broadly expressed in a number of tissues and developmental stages of vertebrates (Wienholds and Plasterk, 2005) and invertebrates (Jagadeeswaran et al., 2010), including *A. mellifera* (Chen et al., 2010; Nunes et al., 2013b). Moreover, several studies reported a wide spectrum of roles for miR-184, such as germline differentiation, axis formation of the egg chamber, anteroposterior patterning and cellularization of the embryo, gastrulation and neuroectoderm formation, apoptosis, and processes involved in the development and differentiation of imaginal discs (head, wing, and eyes) (see Iovino et al., 2009; Li et al., 2011, and references therein). The ecdysone response of miR-184 seen here in pharate-adult honeybees is associated with a period of extensive tissue remodeling, suggesting that miR-184 may play a role in the differentiation of honeybee imaginal disc-derived structures and maintenance of their tissue identities. Interestingly, the EcR mRNA has predicted binding sites for miR-14 (data not shown), and our global gene expression assays revealed a downregulation of this miRNA in bees silenced for *EcR* gene function. These results suggest that in *A. mellifera*, *EcR* gene expression is regulated in a loop-type mechanism involving miR-14, as already demonstrated for *D. melanogaster* (Varghese and Cohen, 2007; for a comprehensive review see Yamanaka et al., 2013).

## Concluding remarks

Our results suggest a differential use of EcR isoforms during the honeybee life-cycle stages. We could show that there is a generally positive *EcR* gene response to slight increases in ecdysteroids, whereas high levels of these hormones are repressive. The EcR knockdown experiments revealed that the expression of several hormone response-related genes (e.g., *kr-h1*) is contingent on a functional ecdysone/EcR receptor complex, thus providing a possible link between JH and ecdysteroid action during preimaginal honeybee development. These knockdown experiments also hightlighted the relevance of a set of miRNAs involved in the regulation of immune response genes and in the general morphogenesis processes during pharate-adult development (e.g., *miR-184* and *let-7* locus genes). Within this framework and on the background of current knowledge on honeybee biology, our results highlight the relevance of the drop in the ecdysteroid pathway function for the appropriate timing in the expression of adult-specific genes, such as the Major Royal Jelly Protein (MRJP) family members.

## Author contributions

Tathyana R. P. Mello, Aline C. Aleixo, Angel R. Barchuk, and Zilá L. P. Simões conceived the project; Tathyana R. P. Mello, Aline C. Aleixo, Daniel G. Pinheiro, Francis M. F. Nunes, Klaus Hartfelder, and Angel R. Barchuk performed the experiments; Tathyana R. P. Mello, Aline C. Aleixo, Daniel G. Pinheiro, Francis M. F. Nunes, Márcia M. G. Bitondi, Klaus Hartfelder, Angel R. Barchuk, and Zilá L. P. Simões analyzed and interpreted the data and drafted the MS. All authors approved the final version of the MS.

## Funding

This study was funded by the Fundação de Amparo à Pesquisa do Estado de São Paulo (FAPESP), grants 2011/03171-5; 2008/1446-4; 2008/10757-3.

### Conflict of interest statement

The authors declare that the research was conducted in the absence of any commercial or financial relationships that could be construed as a potential conflict of interest.
