# Supplementary material for: Developmental regulation of ecdysone receptor (EcR) and EcR-controlled gene expression during pharate-adult development of honeybees (Apis mellifera)
Source: Front Genet. 2014 Dec 22;5:445. doi: 10.3389/fgene.2014.00445 (PMC4273664; doi:10.3389/fgene.2014.00445)
Supplement: Supplementary file 1 [file Table1.PDF]

Table S1. Primers used in the qPCR assays for poly-A^+ RNAs and miRNAs.

**Primer**	**Predicted gene**	**Sense primer**	**Antisense primer**	**Annealing temp (˚C)**	**Fragment length (bp)**
EcRA	*Ecdysone receptor*	CCAACAGCAACAACGGCTAC		60ºC	106
EcRB	*Ecdysone receptor*	ACAGTGTTGCCAACGGTCAC		60ºC	86
EcRA/B	*Ecdysone rec*		AAAGAGCCAGGCTGCGACAA	60ºC	
rpl32	*Ribosomal protein L32*	CGTCATATGTTGCCAACTGGT	TTGAGCACGTTCAACAATGG	60ºC	150
U5 snRNA	*U5 snRNA*	CTCTGGTTTCCCTTCAAATC	ATCAATTGTTCCCCTCCACG	60ºC	74
Ftz-f1	*Ftz-f1*	TCTTCTCCAGATTCGAGTCCA	GAAATGTTTGGCTGGGAAGA	60ºC	119
Vg	*Vitellogenin*	GCAGAATACATGGACGGTGT	GAACAGTCTTCGGAAGCTTG	60ºC	146
AmelCPR14	*AmelCPR14*	CAAGCAATGGGATCAGCCAC	GAAGCCATTCTCGTCGGCTA	60ºC	146
BursA	*bursicon α*	GCGAAAGAGAGGCCAGTGTA	GGCAAATCCAGCAATCTCTT	60ºC	161
P450	*cytochrome P450 6AS5*	GCGACGAGATCTCCCTTTCT	TGCGGTGATAATGGTTCCGT	60ºC	128
Kr-h1	*Ankyrin-repeats protein*	GCGGGAAGTCCTTTGGTTAC	ACCAACCAACTGATGAGTCTGA	60ºC	159
NPC2	*Niemann-Pick disease type C2*	GGTTGCGATACTCTGCCTTG	TGGGAGTGGGTATTGCATGT	60ºC	144
SECP	*Secapin*	GCTGATTTAGTCCCGGAACC	GGCACTATGACTCTACATCTGT	60ºC	98
miR-14		TCAGTCTTTTTCTCTCTCCTA		60ºC	21
miR-125		CCCCTGAGACCCTAACTTGTGA		60ºC	22
miR-100		AACCCGTAGATCCGAACTTGTG		60ºC	21
miR-133		TTGGTCCCCTTCAACCAGCTGT		60ºC	22
miR-3771		TAAAACACAGCGAGTTGCACGAG		60ºC	23
miR-3728		CCATCCGTGGGATTCGTAATTTA		60ºC	23
