# Supplementary material for: Developmental regulation of ecdysone receptor (EcR) and EcR-controlled gene expression during pharate-adult development of honeybees (Apis mellifera)
Source: Front Genet. 2014 Dec 22;5:445. doi: 10.3389/fgene.2014.00445 (PMC4273664; doi:10.3389/fgene.2014.00445)
Supplement: Supplementary file 5 [file Presentation1.PDF]

Figure S1. Relative expression levels of four protein-coding genes (A) and seven miRNAs (B) following *EcR* knockdown. Treatment was as described in Figure 4. Bar-and-whisker plots represent Means; Means±Se; Means±1.96SE for 12 samples, each run as 3 technical replicates. All differences between KD (EcR dsRNA) and C (GFP dsRNA) gene transcription values were statistically significant (Student's *t*-test $P<0.05$).

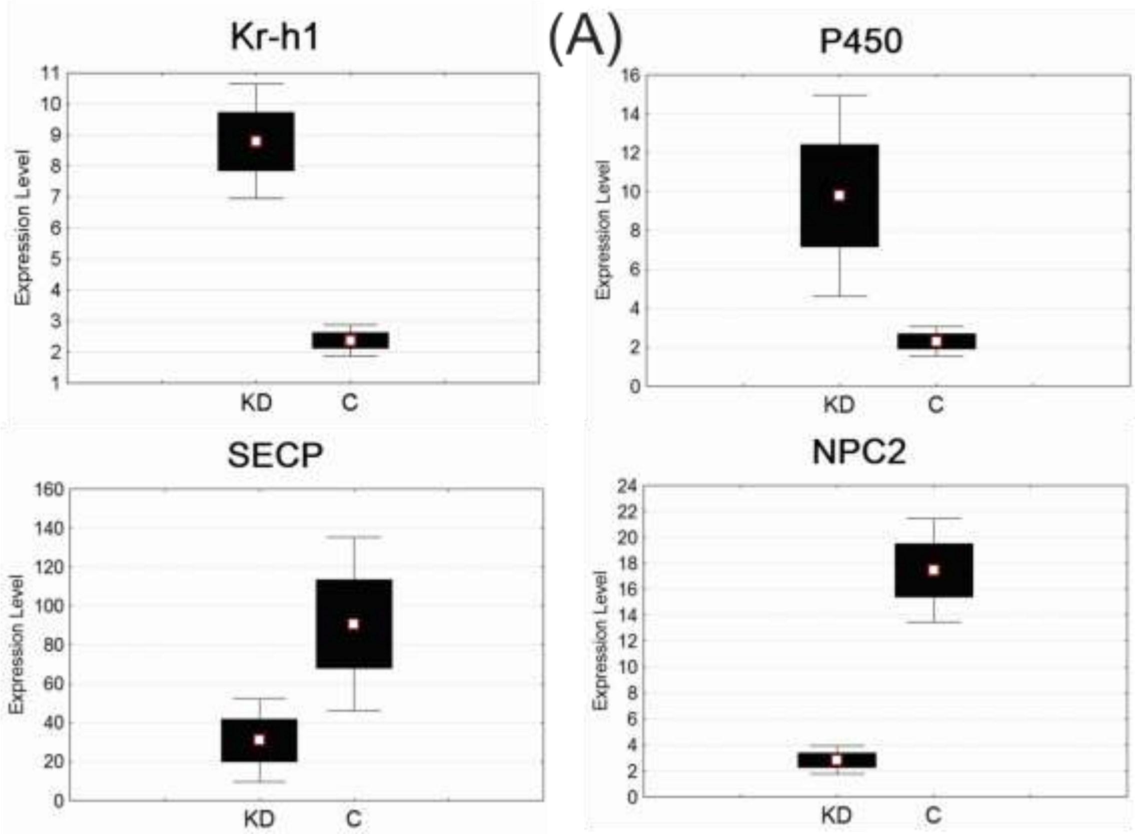

(B)

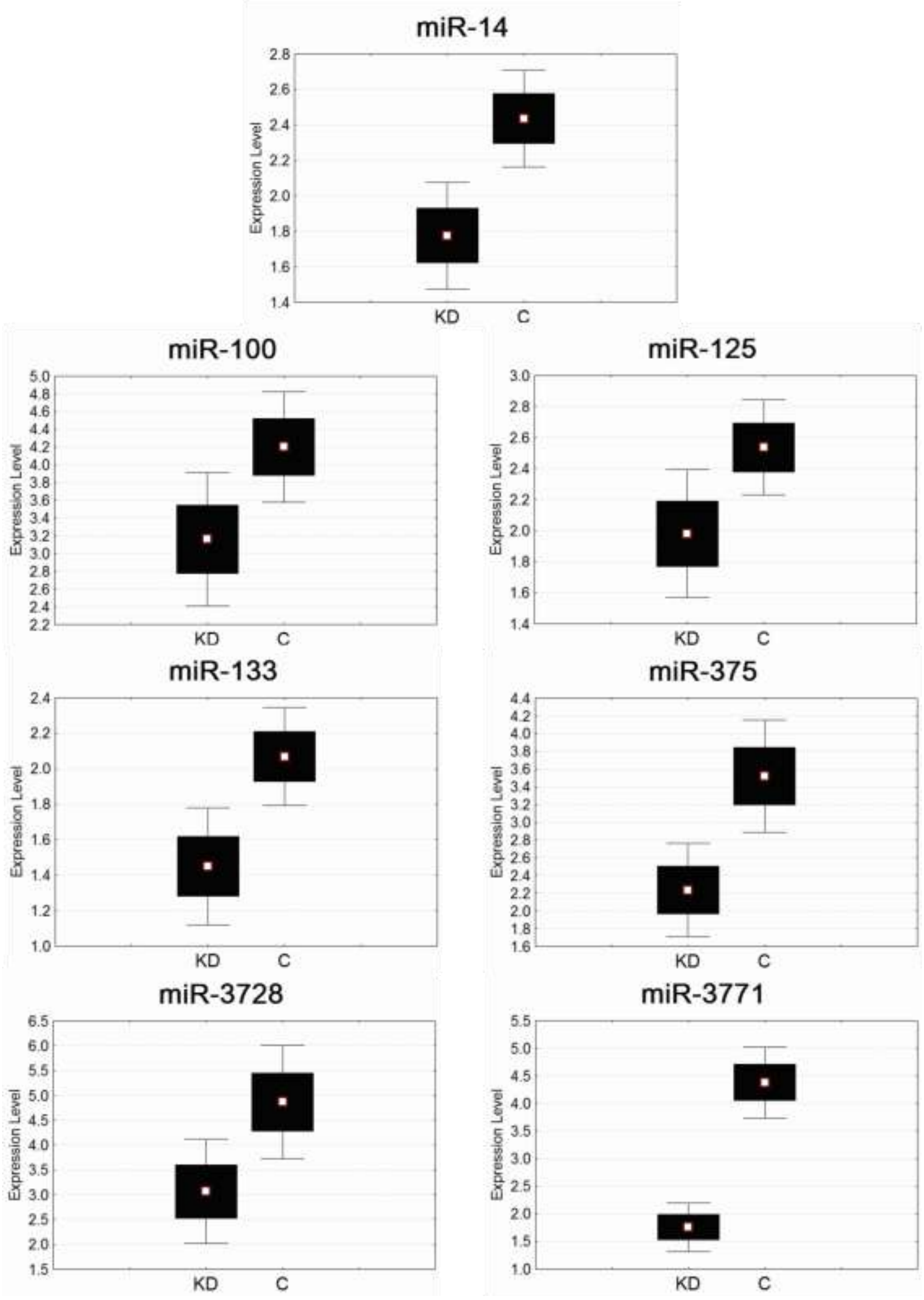
miR-14
Expression Level
KD
C
miR-100
Expression Level
KD
C
miR-125
Expression Level
KD
C
miR-133
Expression Level
KD
C
miR-375
Expression Level
KD
C
miR-3728
Expression Level
KD
C
miR-3771
Expression Level
KD
C
